# Supplementary material for: Soluble Plasma Proteins of Tumor Necrosis Factor and Immunoglobulin Superfamilies Reveal New Insights into Immune Regulation in People with HIV and Opioid Use Disorder
Source: Vaccines (Basel). 2024 May 9;12(5):520. doi: 10.3390/vaccines12050520 (PMC11125794; doi:10.3390/vaccines12050520)
Supplement: Supplementary file 1 [file vaccines-12-00520-s001.zip › vaccines-2983208-supplementary.pdf]

Suppl Table 1

Table S1: Checkpoint multiplex protein panel

**Checkpoint multiplex protein panel 1**	Checkpoint multiplex protein panel 2
1. BTLA (B- and T-lymphocyte attenuator)*<br>2. CD27 (Cluster of differentiation 27)#<br>3. CD28 (Cluster of differentiation 28)*<br>4. CD40 (Cluster of differentiation 40)#<br>5. CD80/B7-1 (Cluster of differentiation 80/B7-1) *<br>6. CD86/B7-2 (Cluster of differentiation 86/B7-2*<br>7. CTLA-4 (cytotoxic T-lymphocyte-associated protein 4)*<br>8. GITRL (Ligand for receptor TNFRSF18/AITR/GITR)#<br>9. GITR (Glucocorticoid-induced TNFR-related protein) #<br>10. HVEM (Herpesvirus entry mediator) #<br>11. ICOS (Inducible T-cell costimulator)*<br>12. LAG-3 (Lymphocyte-activation gene 3)*<br>13. PD-L1 (Programmed death-ligand 1)*<br>14. PD-L2 (Programmed death-ligand 2)*<br>15. PD-1 (Programmed cell death protein 1)*<br>16. TIM-3 (T-cell immunoglobulin and mucindomain containing-3) *<br>17. TLR-2 (Toll like receptor 2) *	1. 4-1BBL/TNFSF9 #<br>2. 5'-NT/CD73 (5'-nucleotidase),<br>3. APRIL (TNFSF13) #<br>4. Arginase-1<br>5. B7-H2/ICOSL (Inducible T cell costimulator ligand*<br>6. B7-H3/CD276*<br>7. B7-H4/VTCN1 (V-set domain-containing T-cell activation inhibitor 1)*<br>8. B7-H5/VISTA (V-set immunoregulatory receptor)*<br>9. B7-H6 (Natural killer cell cytotoxicity receptor 3 ligand) *<br>10. BAFF/BLyS (TNFSF13B) #<br>11. CD25/IL-2Rα (Interleukin-2 receptor alpha)<br>12. CD30/TNFRSF8#<br>13. CD40L (CD40 ligand) #<br>14. CD137/4-1BB (TNFRSF9) #<br>15. CD226/DNAM-1 (DNAX accessory molecule 1)*<br>16. E-Cadherin<br>17. FGL1/Hepassocin (Fibrinogen-like protein 1)<br>18. Galectin-1 (Gal-1)*<br>19. Galectin-3 (Gal-3)*,<br>20. Granulysin*<br>21. Granzyme B*<br>22. IDO1 (Indoleamine 2,3-dioxygenase 1)<br>23. MICA (MHC class I polypeptide-related sequence A)*<br>24. MICB (MHC class I polypeptide-related sequence B)*<br>25. Nectin-2 (PVRL2, CD112)*<br>26. Nectin-4 (PVRL4)*<br>27. OX40/CD134 (TNFRSF4) #<br>28. Perforin*<br>29. PVR/CD155 (Poliovirus receptor cell adhesion molecule) *<br>30. Siglec-7 (Sialic acid-binding Ig-like lectin 7, CD328)*<br>31. Siglec-9 (Sialic acid-binding Ig-like lectin 9, CD329)*

*#TNF superfamily and *Ig superfamily molecules as some additional molecules were determined using Millipore Milliplex Checkpoint Protein Panel 1 and Panel 2 with the Flexmap3D instrument. Biomarker concentrations were determined based on standard curves and expressed in pg/ml.*

Figure Legends for Supplementary Figures:

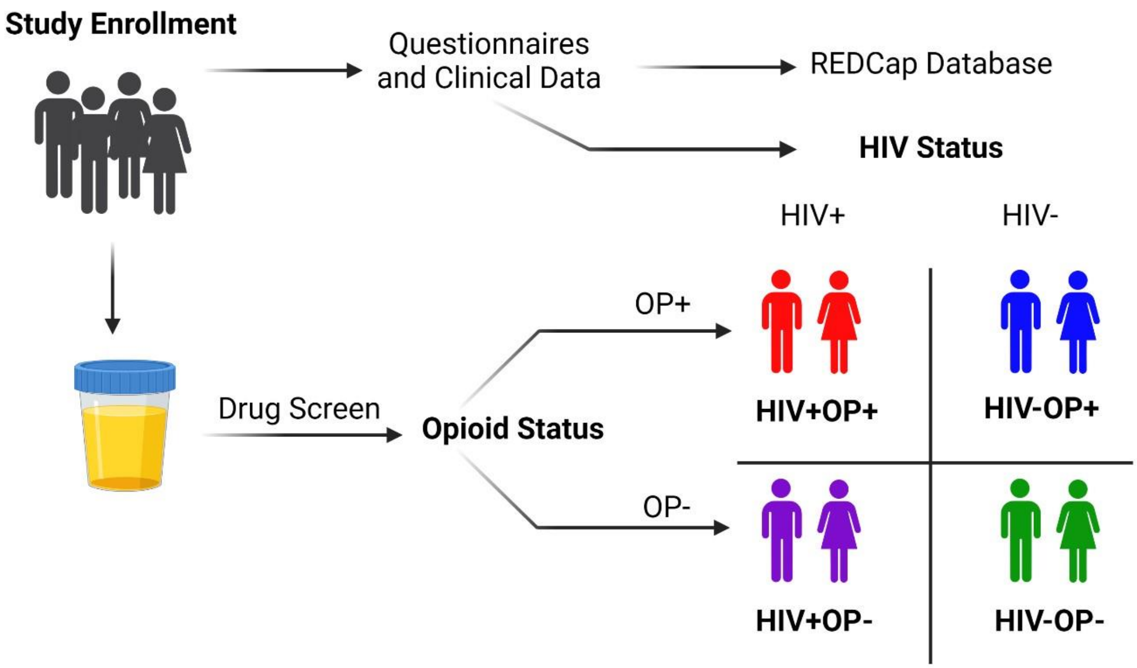

Figure S1: Opioid Immunity Study (OPIS) Study Design**.** (A) Four groups are recruited based on HIV status and opioid use status. Urine samples are collected to test opioid and polydrug use. Red represents HIV+OP+, Blue represents HIV-OP+, Purple represents HIV+OP-, and Green represents HIV-OP-. Participants of all genders are recruited into the study.
Design Figure was created with BioRender.com.

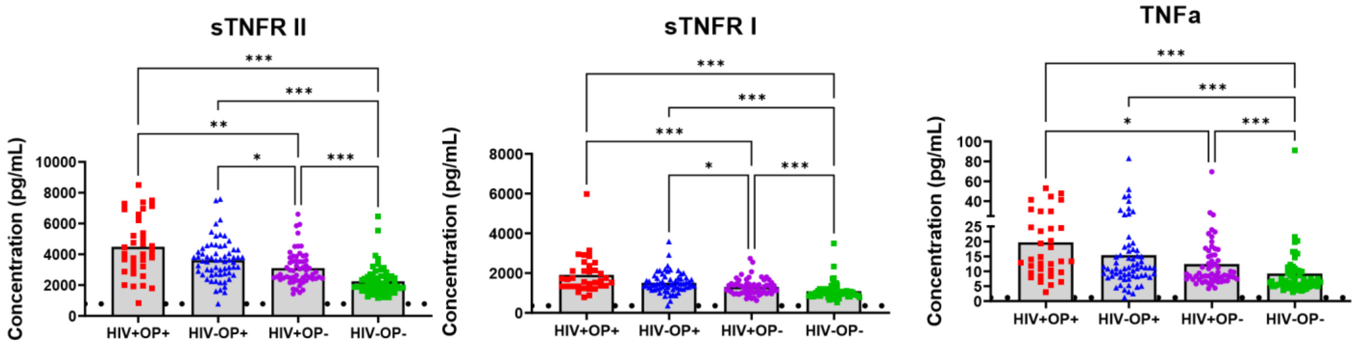

Figure S2: Raw Baseline Concentration of sTNFRII, sTNFRI and TNFa**.** Individual box plots with mean and SEM confidence intervals of sTNFRII, sTNFRI and TNFa. Red represents HIV+OP+, Blue represents HIV-OP+, Purple represents HIV+OP-, and Green represents HIVOP-. Nonparametric Kruskal Wallis test was corrected for multiple comparisons by controlling the FDR (original FDR method of Benjamini and Hochberg). Adjusted p-values: ****p<0.0001, ***p<0.001, **p<0.01, *p<0.05. The limit of detection is indicated with a dotted line.

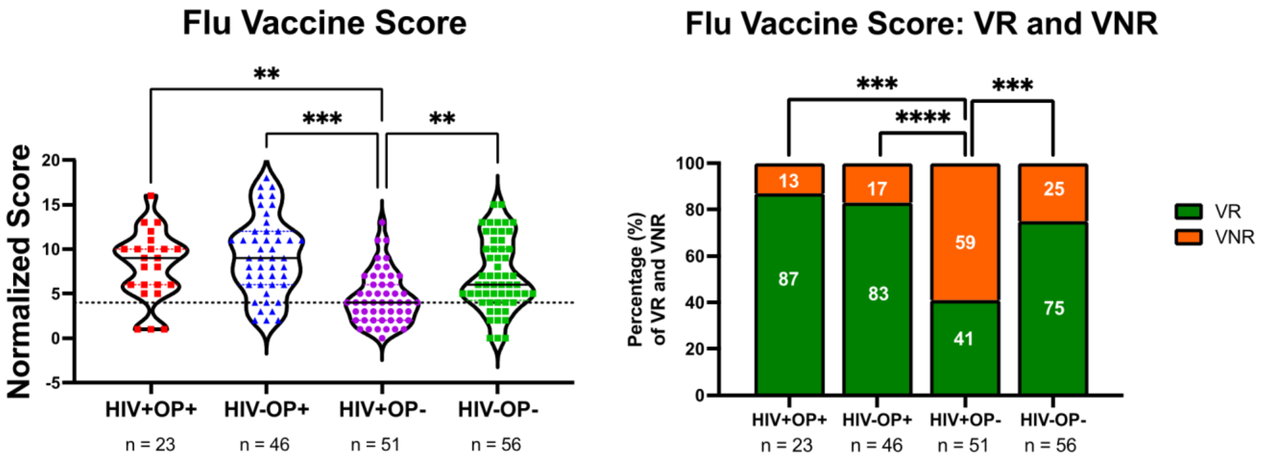

Figure S3: Violin dot plots with the median expression of normalized flu vaccine score. (Non-parametric Kruskal-Wallis test with Dunn's multiple group comparisons. **p<0.01, ***p<0.001). Stacked bar plots displaying percentages of vaccine responders (VR) and vaccine non-responders (VNR) based on normalized flu vaccine scores. Vaccine responders are defined by a vaccine score greater than 4, while people with a vaccine score of less than or equal to 4 were classified as vaccine non-responders (Non-parametric Chi-Square test with Fisher's Exact Test comparison between two groups. **p<0.01), ***p<0.001, ****p<0.0001).
